# Effects of Estrogen Receptor Modulators on Morphine Induced Sensitization in Mice Memory

**Published:** 2015-06

**Authors:** Mahdieh Anoush, Ali Jani, Moosa Sahebgharani, Mohammad Reza Jafari

**Affiliations:** 1 Department of Pharmacology and Toxicology, School of Pharmacy, Zanjan University of Medical Sciences, Zanjan, Iran; 2 Department of Pharmacology, School of Medicine, Tehran University of Medical Sciences, Tehran, Iran; 3 Department of Physiology and Pharmacology, School of Medicine, Zanjan University of Medical Sciences, Zanjan, Iran

**Keywords:** *Estradiol Valerate*, *Raloxifene*, *Step-Down*, *Sensitization*, *Memory*, *Opioids*

## Abstract

**Objective**: In this study, the effects of estradiol valerate and raloxifenea selective estrogen receptor modulator; (SERM) on morphine induced sensitization were examined in mice memory, according to the step-down passive avoidance task.

**Method:** The mice received morphine or estradiol and raloxifene for three days alone or in combination with morphine. After a drug free period of 5 days, the subjects received saline or morphine as pre- training treatments followed by a pre-test saline administration. The memory retrieval was evaluated using step-down passive avoidance test both on the training and test day.

**Results:** The results illustrated that the three- day administration of morphine induced sensitization through the enhancement of memory retrieval (morphine induced sensitization in mice memory). Both the three- day administration of estradiol valerate alone and with morphine (5 mg/kg) restored memory. On the other hand, the three- day administration of raloxifene had no effect on memory retrieval alone, but declined morphine induced sensitization in mice memory.

**Conclusion:** The results of the study indicated that there is an interaction between estrogen receptor modulators and morphine induced sensitization in mice memory.

Large body of evidence indicate that abuse of such drugs as morphine affects neuronal plasticity in brain areas related to motivation and reward (1). Besides, previous studies revealed that morphine and other opioid agents can affect learning and memory (2). The above mentioned effect has been shown both in positive and negative aspects by diverse studies (3, 4). These divergences may be due to different experimental paradigms (2, 3) such as acute or chronic drug administration (2, 4). Many studies have pointed out that acute administration of opioids diminishes learning and memory processes in different types of memory assessment tasks (5-7), and this destruction can be antagonized by naloxone (8-10). Some studies revealed that the pre-training administration of morphine inhibits the acquisition of memory in different paradigms such as y-maze model (11), active or passive avoidance (12) and operant tasks (13). It has been shown that chronic exposure to morphine results in learning impairment in Morris water maze (4). Also, it was reported that frequent exposure to morphine slowed acquisition but did not reduce memory retention in water maze task (2). Repeated administration of morphine pursued by a drug-free status can induce sensitization which in turn results in long-lasting augmentation of morphine behavioral effects (14, 15). The sensitization induced pathways are complex which represents a cascade of events involving either neurotransmitter systems or brain regions such as nucleus accumbens, ventral tegmental area and the hippocampus (16). Behavioral sensitization demonstrated drug-induced neuroadaptive long-term changes in reward-associated pathways in the brain (17).

It has been proved that several effects of acute and chronic exposure to morphine are expressed differently on the basis of gender such as anti-nociception (18), locomotion (19) and development of tolerance and dependence (20). Moreover, according to previous researches, estrogen has been demonstrated to influence learning and memory (21), while its efficacy varies with task study design (22), types of memory (23), and the duration of hormone administration (24).

According to previous reports, estrogen plays a considerable role in induction of acute tolerance to morphine induced analgesia (25). Moreover, it has been reported that morphine-associated contextual memory can be diminished by tamoxifen, and this impairment might be banned by estradiol treatment (26). On the other hand, spinal kappa- and mu-opioid receptor hetero-dimerization can be modified via spinal synthesis of estrogen and simultaneous signaling by membrane estrogen receptors and female-specific spinal morphine antinociception (27). Many studies have been conducted on estrogen and morphine interactions, but there are no reports on the effects of estrogen towards morphine induced sensitization in mice learning. 

The purpose of this study was to evaluate the effects of various doses of estradiol valerate and raloxifene (a selective estrogen receptor modulator; SERM) on morphine induced sensitization in mice memory.

## Material and Methods

*Animals*

Male adult NMRI mice (bred in animal department, School of Pharmacy, with ISO17025 license) weighing 20.2–29.4 g were used in the present study. The animals were housed in a temperature/moisture controlled (22±3°C/45-55% humidity) colony room and were maintained in a 12-h light/dark cycle with free access to food and water, except during experiments. Experiments were done between 10:00 a.m. and 3:00 p.m. Animals were adapted to the laboratory conditions for at least 72 hours prior to experiments. Each treatment group included ten animals. The protocols were carried out according to national guidelines for animal care and use which was approved by the Ethics Committee of the institute.

Drugs and Chemicals:

Morphine sulphate was purchased from Temad (Iran). Estradiol valerate, raloxifene and ultra- filterated sesame oil (as a vehicle for estradiol) were purchased from Iran Hormone Company. Polyethylene glycol 300 (PEG300) was purchased from Merck Schuchardt OHG (Hohenbrunn, Germany).

Morphine sulfate was dissolved in normal saline (0.9%) and estradiol valerate emulsified in sesame oil and normal saline (0.9%). PEG300 was used as a vehicle for raloxifene.

*Passive Avoidance Apparatus*

The passive avoidance apparatus includes a wooden box (30×30×40 cm height), the floor of which is made of 29 parallel stainless steel bars (0.3 cm in diameter, spaced 1 cm apart). A wooden platform (4×4×4 cm) is set in the center of the grid floor. Intermittent electric shocks (1 Hz, 0.5 sec, 50 V DC) were transferred to the grid floor by an insulated stimulator (Panlab LE12106, Spain). A single-trial step-down passive avoidance task was accomplished applying this apparatus. Each mouse was gently placed on the wooden platform. When the mouse stepped-down from the platform and placed all its four paws on the grid floor, then it received electric shock for 15 sec. For establishing the retention test, each mouse was placed on the platform again at 24 h following training and the step-down latency was recorded with a stopwatch. An upper cut-off time of 300 sec was allocated for time recording.

*Experiments*

Experiment 1: This experiment examined morphine induced sensitization in passive avoidance memory. Animals in one control group received 10 ml/kg normal saline subcutaneously (S.C) both in pre-training and pre-test administrations. The other control group received 5 mg/kg (S.C) morphine as pre-training and saline as a pre-test treatment. Three other groups received 5, 10 and 20 mg/kg morphine intraperitoneally (I.P) for three days; after five days of drug-free period, they received 5 mg/kg morphine as pre-training followed by a pre-test administration of saline (10 ml/kg, I.P).

Experiment 2: This experiment assessed the role of estradiol valerate on morphine induced sensitization in learning. In these experiments all animal groups received morphine (5 mg/kg, S.C) as pre-training and saline (10 ml/kg) as pre-test treatment. One set of animal groups received 0.45, 0.9 and 1.8 mg/kg estradiol valerate intraperitoneally (I.P) with a concomitant saline administration (10 ml/kg, I.P) for three days. The other set of groups received 0.45, 0.9 and 1.8 mg/kg estradiol valerate (I.P) with a concomitant morphine administration (5 mg/kg, S.C) for three days.

Experiment 3: This experiment evaluated the role of raloxifene on morphine induced sensitization in learning. In this experiment, animal groups received 10 ml/kg saline or 5 mg/kg morphine (S.C) as pre-training and saline (10 mg/kg) as a pre-test treatment. One set of animal groups received 5, 10 and 20 mg/kg raloxifen (I.P), followed by saline administration (10 ml/kg, I.P) for three days. The other set received 5, 10 and 20 mg/kg raloxifen (I.P) and morphine (5 mg/kg, S.C) for three days.

*Data Analysis*

The step-down latencies were expressed as the median and interquartile range. Data were analyzed using Kruskal–Wallis non-parametric one-way analysis of variance (ANOVA), followed by two-tailed Mann–Whitney U-test completed by a Holm’s Bonferroni correction for the paired comparisons to evaluate the significance of the results. In all statistical evaluations P<0.05 was used as the criterion for statistical significance.

## Results

The results illustrated that pre-training administration of morphine (5 mg/kg) impaired the memory retrieval on the test day compared to the saline-treated group (Mann Whitney U-test, P < 0.001), which was restored in groups which received different doses of morphine for three days (5, 10 & 20 mg/kg) (morphine induced sensitization in memory)

**Fig 1:**
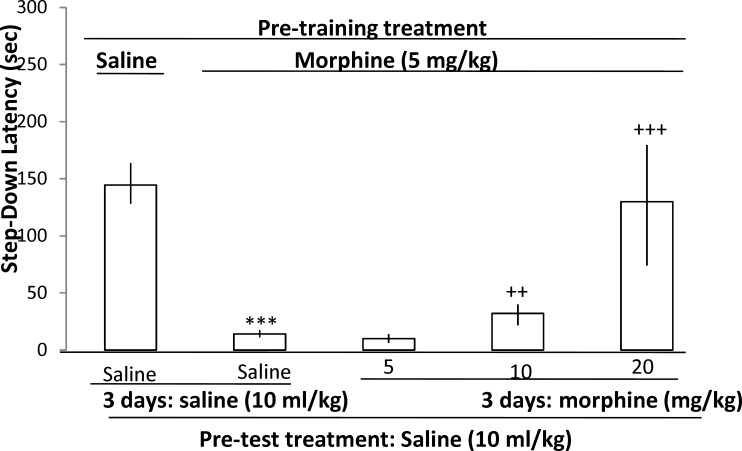
The effect of (3 days different doses of morphine + pre-training saline or morphine

+ pre-test saline) on the step-down latencies compared to relevant control groups. Each value represents the median and quartile of 10 animals.

*** P < 0.001 compared to pre-training and pre-test saline.

++ P < 0.01 and

+++ P < 0.001 compared to pre-training morphine (5 mg/kg) and pre-test saline in figure.

**Fig 2:**
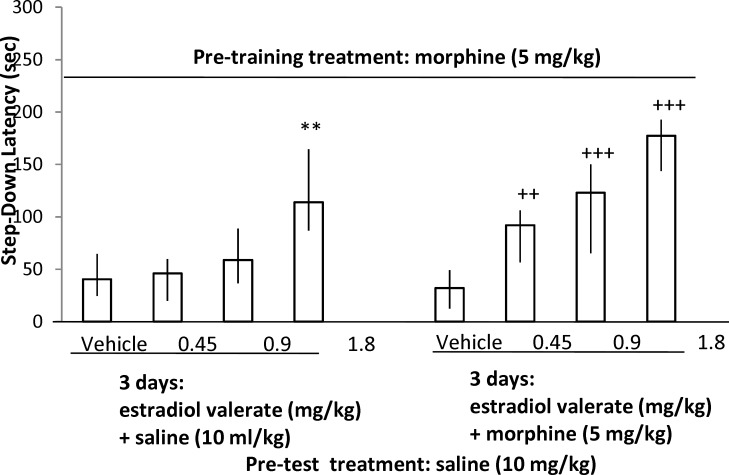
The effect of 3 days administration of different doses of estradiol valerate or estradiol valerate + morphine before the administration of pre-training 5 mg/kg of morphine on the step-down latencies (compared to respective control groups). Each value represents the median and quartile of 10 animals.

** P < 0.01 3 days administration estradiol valerate (1.8 mg/kg) plus saline

+ pre-training morphine (5 mg/kg)

+ pre-test saline compared to the group received vehicle instead of estradiol valerate.

++ P < 0.01 and +++ P < 0.001 3 days administration estradiol valerate plus morphine (5 mg/kg)

+ pre-training morphine (5 mg/kg)

+ pre-test saline compared to the group received vehicle instead of estradiol valerate.

**Fig 3:**
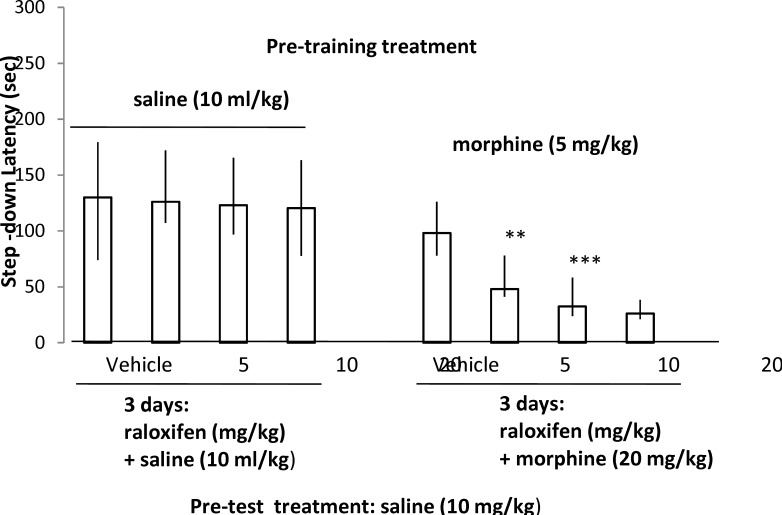
The effect of 3 days administration of different doses of raloxifene or raloxifene + morphine before the administration of pre-training saline or morphine and pre-test saline on the step-down latencies (compared to respective control groups). Each value represents the median and quartile of 10 animals.

** P < 0.01 and

*** P < 0.001 3 days administration of raloxifene plus morphine (20 mg/kg)

+ pre-training morphine (5 mg/kg)

+ pre-test saline compared to the group received vehicle instead of raloxifene.

(Kruskal-Wallis Non-Parametric ANOVA; H (3) = 29.24, p < 0.001, Mann Whitney U-test, P = 0.003 & 0.00003 for 10 and 20 mg/kg of morphine) (Fig.1).

As shown in Figure 2 (the left columns), a- three day administration of estradiol valerate (0.45, 0.9 and 1.8 mg/kg) enhanced memory retrieval which had been impaired by pre-training 5 mg/kg of morphine (Kruskal-Wallis Non-Parametric ANOVA; H (3) = 12.78, p= 0.005). The best result was obtained with 1.8 mg/kg of estradiol valerate (Mann Whitney U-test, P= 0.971, 0.436 and 0.006 for 0.45, 0.9 and 1.8 mg/kg of estradiol valerate respectively). Moreover, in Figure 2 (the right columns), it has been demonstrated that the three- day co-administration of different doses of estradiol valerate (0.45, 0.9 and 1.8 mg/kg) with morphine (5 mg/kg) enhanced the memory retrieving effect of pre-test morphine (5 mg/kg) compared to vehicle + morphine-treated animals (Kruskal-Wallis Non-Parametric ANOVA; H (3) = 24.06, p < 0.001). All doses of estradiol valerate had a significant effect on morphine sensitization learning (Mann Whitney U-test, P = 0.002, 0.0004 and 0.00006 for 0.45, 0.9 and 1.8 mg/kg of estradiol valerate respectively). As shown in Figure 3 (the left columns), the three-day administration of raloxifene (5, 10 and 20 mg/kg) did not affect memory retrieval by saline (Kruskal-Wallis Non-Parametric ANOVA; H (3) = 0.29, p = 0.962). However, the right columns illustrated that the three-day co-administration of raloxifene (5, 10 and 20 mg/kg) with morphine (20 mg/kg) diminished the memory retrieval effect of pre-training 5 mg/kg of morphine (morphine induced sensitization) compared to vehicle-treated group (Kruskal-Wallis Non-Parametric ANOVA; H (3) = 22.87, p < 0.0001). All doses of raloxifene had a significant impairing effect on memory sensitization induced by morphine (Mann Whitney U-test, P = 0.004, 0.00002 & 0.000003 for 5, 10 and 20 mg/kg of raloxifene, respectively).

## Discussion

The results of this study revealed that pre-training administration of morphine (5 mg/kg) impaired the memory retrieval on the test day. Results from previous studies on opioids role in memory are notorious; it has been shown that spatial memory and synaptic plasticity (28) (29) has been diminished by morphine infusion into medial septum. On the contrary, some findings proved that opioids can improve synaptic plasticity in hippocampus (30, 31).

The results obtained from morphine induced sensitization in mice learning illustrated that the three-day administration of morphine (5, 10 and 20 mg/kg) restored memory impairment by pre-training administration of morphine (5 mg/kg). These findings confirm the sensitization in learning induced by morphine which was first introduced in 2000 (15) and later on (1, 32-38). 

Moreover, the results indicated that administration of estradiol valerate for three days instead of morphine, enhanced memory retrieval which was impaired previously by pre-training morphine. Several evidences propose that the most important gonadal steroid hormone (17ß-estradiol) in females may have a positive effect on memory and learning such as motor skills and spatial memory. Potentiating cerebellar plasticity and synapse formation in motor skills have been suggested as mechanisms involved in memory and learning. It has been reported that at least one of the estrogen receptors (alpha) in the hippocampus involves in spatial memory enhancement (39, 40). Latest studies indicated that estrogen enhances spatial reference memory (22) and working memory as well (23). Plasticity in learning and memory occurs primarily in hippocampus, amygdale and cerebral cortex in the brain (41).

On the other hand, the present study showed that co-administration of estradiol valerate with morphine, improved morphine induced sensitization in mice learning (Fig 2). Plenty of studies have been carried out to examine brain regions (1, 33, 34, 42) or drugs which affect morphine induced sensitization in memory (32, 35, 43), but there is no evidence on estrogen’s effect towards the above mentioned phenomenon. Although there is little information on morphine and estrogen interactions, it has been reported that prenatal morphine exposure had altered the performance of adult male and female rats on learning and spatial memory related tasks according to sex differences (44). In addition, post-training or pre-testing injection of estradiol has amplified morphine-induced conditioned place preference (CPP) in a dose-dependent manner (26). Also, a virus injection (iAbeta packaged virus injection) was observed to impair both the spatial memory performance in rats and Morris water maze test ranks in mice, which were restored by morphine administration and estradiol release in hippocampal neurons (45).

In the last experimental set of this study, the three- day I.P injection of raloxifene alone represented no significant effect on memory retrieval. According to previous studies, the effects of raloxifene on memory are paradoxical. In other words, it has been demonstrated that raloxifene did not damage cognition or affect mood in postmenopausal women (46), but brain activation patterns upon visual encoding in postmenopausal women were altered by the drug (47). It has been illustrated that overall cognitive scores in osteoporotic postmenopausal women have not been affected by the three-year administration of raloxifene (48). In this regard, raloxifene did not improve spatial working memory in aged monkeys despite many years of estrogenic deficit (49) and had no effect on dendritic branching throughout hippocampal development in vitro (50). Also, in ovariectomized rats cognitive performance had not been increased by raloxifene which was evaluated by acquisition of a simple spatial memory task (51). Besides, the chronic administration of raloxifene did not adjust cognitive variables in menopause women (52). However, it has been shown that raloxifene significantly raised neuronal outgrowth of hippocampal neurons within a narrow dose range but did not support the outgrowth of basal forebrain or cortical neurons (53). Raloxifene therapy in healthy elderly men improved brain activation in areas spanning a number of different cognitive domains which may relate to effects on attention as well as different types of memory (54). The mechanism of this effect is increased excitement during initial encoding with downstream effects on brain function during information retrieval (55). Moreover, raloxifene treatment had a significant affirmative effect on both memory deficit and the rate of recovery for the bilateral tactile removal test; also, it had a significant enhancement in the acquisition of working memory in animals (56). Although the drug did not demonstrate a negative effect on cognitive functioning in patients with breast cancer (57), it significantly improved verbal memory in postmenopausal women compared to placebo (58, 59). It has been revealed that raloxifene is capable of either improving prefrontal cortex-related cognitive performance or modulating prefrontal cortex morphology in ovariectomized rats (60). Recently, it was reported that raloxifene administration not only improved verbal memory at lower doses, but also produced a decrease in the risk of mild cognitive impairment at higher doses and lowered the risk of Alzheimer's disease in postmenopausal women (61).

In this study, a three- day protocol of co-administrating raloxifene with morphine diminished morphine induced sensitization in mice learning in contrast to estradiol. There are little data regarding raloxifene (or SERM) and morphine interaction particularly memory related phenomena. In addition, it is revealed that tamoxifen was able to disturb consolidation and retrieval of morphine-associated contextual memory and this impairing effect might be inhibited by estradiol treatment (26). Also, it has been observed that raloxifene did not affect morphine withdrawal induced hyperthermia in ovariectomized rats which was on the contrary to the 17 alpha-Ethinyl estradiol (EE) (62). In the same model, the effect of EE in the morphine-dependent model of hot flush and on body weight fluctuations were reduced by fulvestrant (as a full antagonist of estrogen receptors) (63). In addition, recently, it has been shown that ovariectomy can affect the sensitivity to morphine induced antinociception of neuropathic pain and it can also change K+-Cl- cotransporter 2 (KCC2) protein level in the spinal dorsal horn in Sprague-Dawley rats (64). Moreover, it was revealed that in both male and female rats, expression of androgen and estrogen receptors in the periaqueductal gray (PAG) and the descending pathway driving pain inhibition may modulate pain and morphine potency (65). Also, it has been demonstrated that an estrogen-sensitive mechanism may alter the excitatory amino acid release in the nucleus accumbens and this phenomenon play a role in the morphine analgesia and tolerance (66). Furthermore, it has been shown that opioids exert important effects on plasma and CNS sex hormone levels (67). The observed results in the present study might be related to alterations of cerebral estrogen concentration or other mechanisms like post receptor signaling interactions. 

## Limitation

The present study was done with a SERM (raloxifene) which a full estrogen receptor antagonist like fulvestrant was better than instead.

## Conclusion

The results of the present study revealed an interaction between estrogen receptor modulators and morphine induced sensitization in mice learning.
